# Targeting GBM with an Oncolytic Picornavirus SVV-001 alone and in combination with fractionated Radiation in a Novel Panel of Orthotopic PDX models

**DOI:** 10.1186/s12967-023-04237-w

**Published:** 2023-07-06

**Authors:** Huiyuan Zhang, Yuchen Du, Lin Qi, Sophie Xiao, Frank K. Braun, Mari Kogiso, Yulun Huang, Frank Huang, Aalaa Abdallah, Milagros Suarez, Sekar Karthick, Nabil M. Ahmed, Vita S. Salsman, Patricia A. Baxter, Jack M. Su, Daniel J. Brat, Paul L. Hellenbeck, Wan-Yee Teo, Akash J. Patel, Xiao-Nan Li

**Affiliations:** 1 Texas Children’s Cancer Center, Houston, TX USA; 2 Laboratory of Molecular Neuro-Oncology, Texas Children’s Hospital, Baylor College of Medicine, Houston, TX 77030 USA; 3 Department of Pediatrics, Ann & Robert H. Lurie Children’s Hospital of Chicago, Lurie Children’s Hospital of Chicago, Chicago, IL 60611 USA; 4 Department of Pharmacology, School of Medicine, Sun Yat-sen University, Guangzhou, 510080 Guangdong China; 5 Division of Experimental Hematology and Cancer Biology, Brain Tumor Center, Department of Pediatrics, Cincinnati Children’s Hospital Medical Center, University of Cincinnati College of Medicine, Cincinnati, OH 45229 USA; 6 Pediatric Brain Tumor Research Office, Cancer and Stem Cell Biology Program, SingHealth Duke-NUS Academic Medical Center, Humphrey Oei Institute of Cancer Research, National Cancer Center Singapore, Duke-NUS Medical School, 169610 Singapore, Singapore; 7 Department of Pathology, Northwestern University Feinberg School of Medicine, Chicago, IL 60611 USA; 8 Seneca Therapeutics, Inc, Blue Bell, PA 19422 USA; 9 Department of Neurosurgery, Baylor College of Medicine, Houston, TX USA; 10 Robert H. Lurie Comprehensive Cancer Center, Northwestern University, Chicago, IL 60611 USA; 11 Program of Precision Medicine PDOX Modeling of Pediatric Tumors, Department of Pediatrics, Ann & Robert H. Lurie Children’s Hospital of Chicago, Northwestern University Feinberg School of Medicine, Chicago, IL 60611 USA; 12 Shenzhen Key Laboratory for Systems Medicine in Inflammatory Diseases, School of Medicine, Sun Yat-sen University, Shenzhen, Guangdong, China; 13 Department of Neurosurgery, Dushou Lake Hospital, Soochow University Medical School, Suzhou, Jiangsu, China; 14 Jan and Dan Duncan Neurological Research Institute, Texas Children’s Hospital, Houston, TX USA; 15 Department of Otolaryngology-Head and Neck Surgery, Baylor College of Medicine, Houston, TX USA; 16 Dan Duncan Cancer Center, Baylor College of Medicine, Houston, TX USA

**Keywords:** Patient-derived orthotopic xenograft, Glioblastoma, Oncolytic virus, SVV-001, Therapeutic efficacy

## Abstract

**Background:**

Animal models representing different molecular subtypes of glioblastoma multiforme (GBM) is desired for developing new therapies. SVV-001 is an oncolytic virus selectively targeting cancer cells. It’s capacity of passing through the blood brain barrier makes is an attractive novel approach for GBM.

**Materials and methods:**

23 patient tumor samples were implanted into the brains of NOD/SCID mice (1 × 10^5^ cells/mouse). Tumor histology, gene expression (RNAseq), and growth rate of the developed patient-derived orthotopic xenograft (PDOX) models were compared with the originating patient tumors during serial subtransplantations. Anti-tumor activities of SVV-001 were examined in vivo; and therapeutic efficacy validated in vivo via single *i.v.* injection (1 × 10^11^ viral particle) with or without fractionated (2 Gy/day x 5 days) radiation followed by analysis of animal survival times, viral infection, and DNA damage.

**Results:**

PDOX formation was confirmed in 17/23 (73.9%) GBMs while maintaining key histopathological features and diffuse invasion of the patient tumors. Using differentially expressed genes, we subclassified PDOX models into proneural, classic and mesenchymal groups. Animal survival times were inversely correlated with the implanted tumor cells. SVV-001 was active in vitro by killing primary monolayer culture (4/13 models), 3D neurospheres (7/13 models) and glioma stem cells. In 2/2 models, SVV-001 infected PDOX cells in vivo without harming normal brain cells and significantly prolonged survival times in 2/2 models. When combined with radiation, SVV-001 enhanced DNA damages and further prolonged animal survival times.

**Conclusion:**

A panel of 17 clinically relevant and molecularly annotated PDOX modes of GBM is developed, and SVV-001 exhibited strong anti-tumor activities in vitro and in vivo.

**Supplementary Information:**

The online version contains supplementary material available at 10.1186/s12967-023-04237-w.

## Background

Glioblastoma (GBM) is the most aggressive primary brain tumor in adults. With a median survival of 15 months from diagnosis, this disease is uniformly fatal [1]. Development of chemotherapies has been difficult due to low penetration of the blood–brain barrier. Novel therapeutic strategies are desperately needed to improve patients’ quality-of-life and increase their survival [2]. Most of the current preclinical cancer models are often unable to recapitulate tumor heterogeneity or predict patient response to treatment. Further, gaining a better understanding of the molecular characteristics of individual GBMs is necessary for the development of individualized therapy strategies. This task requires sufficient tumor material for analysis and potentially therapy response prediction methods. Clinically relevant and molecularly accurate animal models of GBM are highly desired since tumor material can be propagated for further studies in an in vivo environment, while maintaining intra-tumoral heterogeneity as well as key genomic aberrations [3, 4]. Direct orthotopic implantation of patient tumor cells into the cerebrum of immunocompromised mice represent a reliable approach to generate clinically relevant animal models. These patient-derived orthotopic xenograft (PDOX or orthoptic PDX) models provide a much needed translational platform to understand cancer biology, identify predictive biomarkers for therapy outcome, and evaluate new therapeutic strategies [5].

Oncolytic virotherapy is a unique approach where naturally occurring or genetically enginerred viruses that selectively or preferentially infect and/or replicate in tumor cells are used to kill the cancer cells. Over the past few years, a number of promising oncolytic viruses have demonstrated anti-glioma activities in both preclinical mouse model and early-phase clinical trials [6]. Seneca Valley Virus 001 (SVV-001) is a single-stranded RNA virus belonging to the *Picornaviridae* family that replicates through an RNA intermediate and does not integrate into the host genome. SVV-001 rapidly enters tumor cells and has been shown to induce rapid tumor cell killing in several in vitro and in vivo models [7, 8]. Exposure to SVV-001 does not appear to be prevalent in the human population or cause any harmful disease [9]. More importantly, we have shown that SVV-001 can penetrate through the BBB in medulloblastoma and pediatric GBM PDOX models to selectively kill tumor cells while sparing normal brain cells [10, 11]. These features make SVV-001 an attractive therapeutic agent for GBM. Since SVV-001 as single agent did not eliminate recurrence in PDOX models of pediatric GBM, we hypothesize that combination with fractionated radiation would further enhance its therapeutic efficacy, as has been observed with other oncolytic virotherapies [12-14].

Recognizing the need of clinically relevant animal models the development of new therapies for gliomas [15], we report here our effort of developing PDOX models of GBM through direct injection of 23 fresh surgical specimens of adult malignant gliomas into the brains of Rag2/Severe Combined Immuno-deficient (SCID) mice. The established xenograft tumors were strictly subtransplanted in vivo in mouse brains and examined for their replication of histopathology and molecular abnormalities of the original patient tumors. Taking advantage of this unique panel of PDOX models, we further examined the antitumor efficacy of SVV-001 acting alone and in combination with fractionated radiation in adult gliomas in vivo following the initial screening in vitro. Our goal is to establish preclinical rational for the initiation of clinical trials of SVV-001 in GBM.

## Materials and methods

### Patient tumor tissue for implantation

Fresh patient tumor tissues were collected from Baylor St Luke’s Hospital (n = 18) and Methodist Hospital (n = 4; Table 1) following Institutional Review Board (IRB) approved protocols after obtaining consent from the patients or family. Xenograft cells from tumor V0914 was established from an anaplastic glioma [16].

**Table 1:** Summary of clinical information and intra-cerebral xenograft development of adult gliomas

No.	Tumor ID	Age/gender	Race/ethnicity	Tumorigenicity at passage I	Passages in vivo
1	K012	43/F	AA	3/4	5
2	K014	70/M	W	6/7	4
3	K023	36/M	AA	4/5	5
4	K024	59/M	W	10/10	4
5	K031	70/M	W	3/7	2
6	K033	66/M	H	5/8	3
8	K035	70/M	W	8/9	5
9	K038	64/M	AA	4/5	4
10	K045	60/M	W	6/7	3
11	K053	72/M	W	1/5	3
12	K060	56/F	W	7/10	3
13	K064	58/F	W	9/9	4
14	A0313	*	*	2/4	5
15	A46	65/F	A	5/5	5
16	A128	83/M	*	3/8	2
17	A129	70/M	*	4/7	2
18	V0914	50/M	*	8/10	5
19	K019	58/M	W	0/5	–
20	K025	45/M	H	0/5	–
21	K032	80/M	W	0/5	–
22	K046	64/F	W	0/5	–
23	K054	75/F	H	0/10	–

### Establishment of transplantable orthotopic PDX mouse models of GBM

The Rag2/SCID and NOD/SCID mice were bred and housed in a specific pathogen-free animal facility at Texas Children’s Hospital. Mice between 12 and 16 weeks of both sexes were used for this study. All the experiments were conducted following a protocol approved by the Institutional Animal Care and Use Committee (IACUC). PDOX models were established through direct injection of fresh surgical specimens into the right cerebrum (GBM, *n* = 23) of the Rag2/SCID mice, which breeds very well and tolerate intra-cranial tumor implantation in our previous studies [17-25], and subtransplanted strictly in vivo in mouse brains following our surgical protocol described previously [26]. Briefly, fresh surgical specimens were mechanically dissociated or trypsinized shortly after tumor removal. After the cell suspensions were passed through 40-µm cell strainers, the live tumor cells as single cells and small clumps (∼5–10 cells) were counted with trypan blue staining. Tumor cells (1 × 10^5^) were then suspended in 2 µL of culture medium, stored at 4 °C and injected into mouse brains 1 mm to the right of the midline, 1.5 mm anterior (for intracerebral tumors) to the lambdoid suture, and 3 mm deep via a 10-µL 26-gauge Hamilton Gastight 1701 syringe needle. The animals were monitored every day until they developed signs of neurological deficit or became moribund, at which time they were sacrificed following our approved animal protocol. The xenograft tumor was then harvested and dissociated into single cells, then directly re-transplanted for expansion in later serial generations by the same procedure or suspended in cryopreservation medium and stored in liquid nitrogen. The frozen tumor cells were later thawed and used for experiments including re-transplantation and expansion. After the tumor tissue had been passaged three times or more and histopathological examination confirmed the PDOX to be a growing human tumor, we considered the PDOX models as ‘established’. Mice that did not develop tumor mass over one year after engraftment were euthanized for histopathologic examination of tumor formation. Unlike Rag2/SCID mice that are sensitive ionizing radiation, NOD/SCID can better tolerate radiation induced damages and survive fractionated radiation therapy as we shown recently [27, 28]. They are therefore prioritized for in vivo therapies involving radiation.

### Histological and immunohistochemical (IHC) staining

For morphological characterization, sample tissues were formalin-fixed, paraffin-embedded, sliced into 5-µm sections, and subjected to standard hematoxylin and eosin (H&E) staining. IHC staining was performed as we described previously [18-20, 24, 29]. IHC staining was performed using a Vectastain Elite kit (Vector Laboratories, Burlingame, CA, USA). Primary antibodies included mouse monoclonal antibodies or rabbit polyantibodies against Ki67 (ab833-500, 1:50; Abcam Inc.), Cow Glial Fibrillary Acidic Protein (GFAP, M0761, 1:100; DAKO Corp.), human-specific mitochondria (MAB1273, 1:50; Chemicon International Inc.), p53 (sc-6243, 1:100; Santa Cruz Biotechnology, Inc.), Vimentin (VMT, M0725, 1:200; DAKO Corp.), Anti-von Willibrand Factor (vWF, AB7356, 1:500; Millipore crop), SVV-001 capsid protein (2A9, 1:200; Neotropix), rH2AX (2577, 1:100; Cell Signaling Technology Inc.), Caspase-3 (9662, 1:50; Cell Signaling Technology Inc), PARP (P248, 1:1000; sigma-aldrich, Inc.), ANTXR1 (OAAB04785, 1:200; Aviva Systems Biology Corp.). The slides were blocked with mouse-on-mouse blocking solution from the kit for 1 h, followed by incubation with the primary antibodies at 4 °C overnight. The appropriate biotinylated secondary antibodies (1:200) were subsequently applied and incubated at room temperature for 30 min, and the final signal was developed using the 3,3′-diaminobenzidine (DAB) substrate kit for peroxidase. Hematoxylin (Vector Laboratories, Burlingame, CA, USA) was used for nuclear counterstaining. Slides were observed and photographed on the Nikon Eclipse microscope at 400× magnification.

### RNA-seq and molecular sub-classification

RNAseq libraries for transcriptome analysis were prepared using the TruSeq RNA Sample Preparation Kit (Illumina) and Agilent Automation NGS system per manufacturers’ instructions. Sample prep began with 1 µg of total RNA from each sample. Poly-A RNA was purified from the sample with oligo dT magnetic beads, and the poly(A) RNA was fragmented with divalent cations. Fragmented poly-A RNA was converted into cDNA through reverse transcription and were repaired using T4 DNA polymerase, Klenow polymerase, and T4 polynucleotide kinase. 3′ A-tailing with exo-minus Klenow polymerase was followed by ligation of Illumina paired-end oligo adapters to the cDNA fragment. Ligated DNA was PCR amplified for 15 cycles and purified using AMPure XP beads. After purification of the PCR products with AMPure XP beads, the quality and quantity of the resulting transcriptome libraries were analyzed using an Agilent Bioanalyzer High Sensitivity chip. RNAseq data has been deposited to GEO (access number: GSE207886). To determine GBM molecular subtypes, two strategies using Verhaak’s 840 gene-set [30] and our previously published TCGA-derived 500 gene classifiers [31] to perform hierarchical clustering and silhouette plot analysis.

### Short Tandem repeat (STR) profiling

Each tumor DNA sample was subjected to STR profiling performed by Guardian Forensic Sciences as we described previously [32]. The GenePrint24 System for STR profiling (Promega, Cat#B1870) was used to amplify 0.05 ng of template DNA using RotorGene Q instrument followed by profiling of samples with Applied Biosystems ABI 310 Genetic Analyzer and data interpretation by forensic biologists. Only samples deemed not misidentified and free of contamination were used in this study.

### The oncolytic virus SVV-001

SVV-001 (1 × 10^11^ vp/mL) and genetically engineered SVV-GFP (1 × 10^12^ vp/mL), which expresses green fluorescent protein (GFP), were obtained from Neotropix (Seneca Therapeutics) Inc. SVV-GFP has the homogeneous tropism as the parent SVV-001 but with decreased cell lysis activities [7, 10, 11, 33-37]. For in vitro treatment, SVV-001 and SVV-GFP were diluted into serum-based Dulbecco’s modified Eagle’s medium for primary cultured monolayer cells and serum-free CSC growth medium containing human recombinant basic fibroblast growth factor (bFGF) and epidermal growth factor (EGF) (50 ng/mL, R&D Systems) for the growth of neurospheres. For in vivo treatment, SVV-001 was diluted with phosphate buffered saline (PBS) and administered through a single tail vein injection at 1 × 10^11^ viral particle (vp)/kg 2–4 weeks post tumor implantation.

### In vitro analysis of viral infection

Cells were seeded at 5,000/well in 96-well plates and cultured overnight. Following the addition of SVV-GFP at multiplicity of infection (MOI) of 20, 200, and 2000 [10, 34, 35], cells were imaged under fluorescence microscopy to detect the expression of GFP at 1, 2, 3 and 7 days and quantitated with flow cytometry. Dead cells were gated out by propidium iodide staining (0.5 µg/mL).

### Cell viability and cytotoxicity assay

Primary cultured xenograft GBM cells were seeded in 96-well clear-bottom plates in quadruplicates. One day after the cells settled, treated with or without SVV-001 at different multiplicities of infection (MOI) of 0.5–25. Cell viability was checked with CCK-8 (Dojindo Molecular Technologies) at 1, 3, 7 and 14 days, as described previously [10, 35].

### Fluorescence-activated cell sorting of CD133^+^/CD15^+^and CD133^−^/CD15^−^ GBM cells

GBM cells were labeled with phycoerythrin (PE)-conjugated monoclonal antibodies against human CD133 (CD133/2-PE, Milteny Bio) and FITC-conjugated monoclonal antibodies against human CD15 (CD15-FITC, Milteny Bio) at 4 °C for 10 min per manufacturer’s instructions, as we described previously [18]. Cells were then washed and resuspended in a stem cell growth medium consisting of Neurobasal media (Invitrogen), N2 and B27 supplements (0.5× each; Invitrogen), human recombinant bFGF and EGF (50 ng/mL each; R&D Systems) [3], penicillin G, and streptomycin sulfate (1:100; GIBCO-Invitrogen). CD133^+^/CD15^+^, CD133^+^, CD15^+^and CD133^−^/CD15^−^ cells were then flow-sorted with BD Aria Fusion. Dead cells were excluded by propidium iodide (PI) staining [18].

### Treatment of PDOX tumors with SVV-001 in combination with fractionated radiation

SVV-001 (1 × 10^11^ vp/kg) was resuspended in 0.1 mL PBS and administered through a single tail vein injection in 2 GBM mouse models at 3 or 4 weeks after tumor injection. Body weights were monitored weekly as a surrogate indicator of SVV-001 systemic side effects. Mice were randomly assigned to 4 groups; (1) untreated control (*n* = 10); (2) SVV-001 treatment (1 × 10^11^ vp/kg) on day 21 after tumor injection (*n* = 10); (3) radiation treatment (total of 10 Gy over 5 days, from day 14 to day 21 after tumor injection) (*n* = 10) (4) SVV-001 treatment (1 × 10^11^ vp/kg) on day 21 after tumor injection combined with radiation (*n* = 10). The tumor part of the brain was delivered radiation using a self-contained X-ray system (RS-2000 Pro Biological Irradiator). During XRT, mice were placed in a customized lead cover to shield the body to allow radiation to be delivered directly to the brain tumor [27]. The total radiation dose administered was 10 Gy at a clinically relevant 2 Gy/fraction schedule on five consecutive days. Mice were sacrificed when they exhibited symptoms indicative of significant compromise to neurologic function and/or a greater than 20% body weight loss. The whole brains were removed and fixed in formalin and embedded in paraffin for histopathological analysis. Animal survival was defined as the time taken from tumor injection until euthanasia and analyzed through log-rank analysis. To study the biological changes caused by SVV-001, we allowed the injected xenograft cells in a separate group to grow for ∼8 weeks to form tumors 8–10 mm in diameter before being treated with SVV-001 as described. Mouse brains were then removed at 1, 3, and 7 days (*n* = 2–3 per time point) after virus injection and examined.

### Statistical analysis

In vitro cytotoxic effects of SVV-001 and changes of SVV-GFP infectivity were analyzed through *t*-test. Differences in animal survival times were analyzed with a log-rank analysis using SigmaPlot 14 (Systat Software) after the animals were randomly assigned to control and testing groups. *P* values <0.05 was determined as significant.

## Results

### Formation and serial subtransplantation of PDOX tumors in the brains of SCID mice

**Fig. 1:**
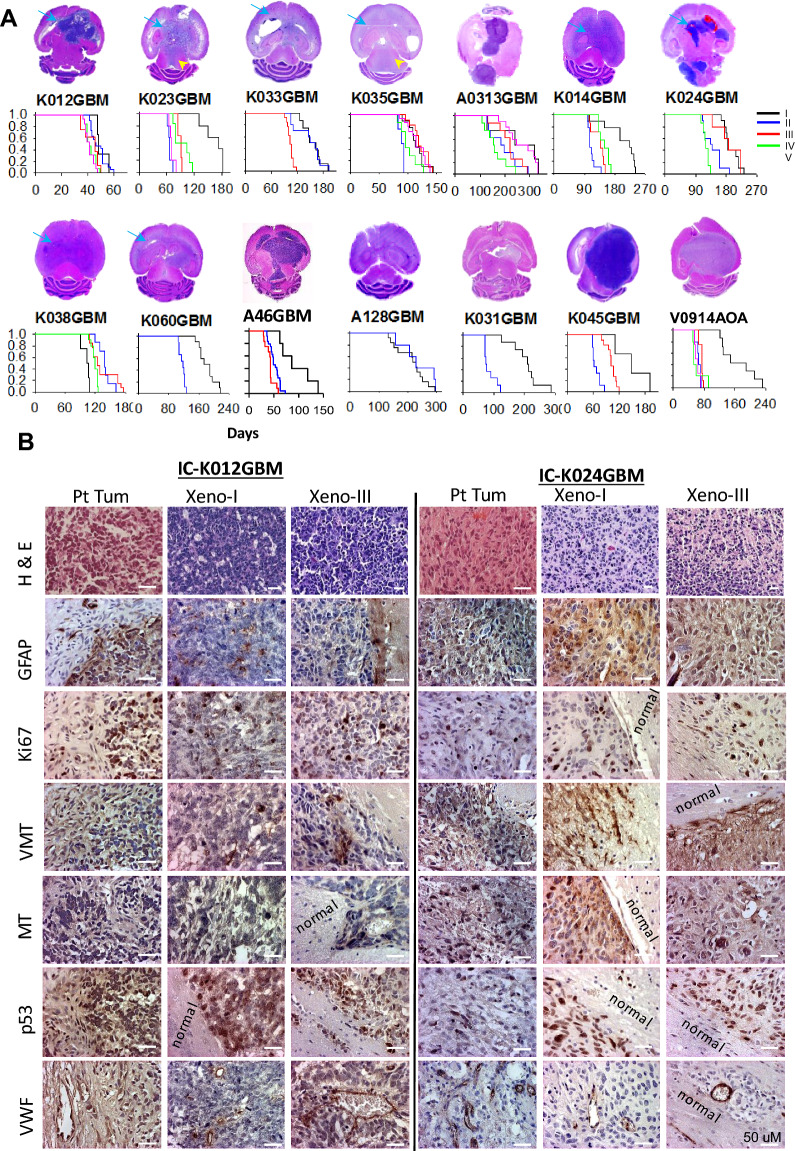
Gross and microscopic images of PDOX models of GBM. **a **Representative images of H&E stained whole mouse brain cross-sections showing intra-cerebral xenograft tumors as well as the invasion into the opposite side (*blue arrow*) and down to the brain stem via CSF (*arrowhead*). Animal survival time during serial subtransplantations (up to passage V) were shown under each tumor image. **b **Comparison of histological and immunohistochemical features between patient tumor and their matching PDOX tumors from passage I and III. In addition of H&E staining, glial marker (GFAP), cell proliferation (Ki-67), human-specific intermediate filament vimentin (VMT) and mitochondria (MT), as well as tumor suppressor gene P53 and tumor micro-blood vessel endothelial cells von Willebrand Factor (vWF) were shown and compared with normal (*Normal*) tissues. (Scale bar: 50 µM)

A total of 23 surgically removed tumor tissues from 14 male and 7 female patients aged between 43 and 83 were engrafted in Rag2/SCID mice (Table 1) at 1 × 10^5^ viable cells per mouse. The animals were monitored daily following IACUC approved protocol and euthanized when they developed signs of neurological deficit or became moribund. Tumor formation was confirmed in 17/23 (73.9%) tumors implanted. Grossly, the mouse brains were invariably enlarged, which, when sectioned, often reveal a huge intracerebral xenograft tumor (Fig. 1a). Animal survival times starting from tumor implantation ranged from 35 to 247 days (149.3 ± 57.6). When comparing the tumorigenic GBMs with those failed to form xenografts, no significant differences were observed in age. Given the sexually dimorphic nature of GBM [38, 39], we also examined the impact of patient sex on tumorigenicity. Among the 22 patient tumors with gender information, PDOX formation was confirmed in 12/15 male and 5/7 female patients. Cha square analysis showed a statistic of 0.1997 and *P* = 0.654, indicating no significant differences. The average age of patients with tumorigenic tumors was 63.2 years, compared with 65.2 years in those did not form PDOX, and there were no statistical differences (*P* = 0.77, *t*-test). Among the 17 tumorigenic GBMs with self-reported racial/ethnic information, there were eight models derived from white/Caucasian, 3 from African American, 2 from Hispanic White, and 1 from Asian.

When the growth of xenograft tumor was confirmed, we harvested the xenograft tumors from donor mice and re-transplanted tumor cells into the brains of recipient mice. All animals were injected with 1 × 10^5^ tumor cells at the subsequent passages and developed intracerebral (IC) tumors. To determine whether repeated subtransplantations would cause major changes of tumor growth rate, we examined the animal survival times using log-rank analysis and pairwise comparison. As we have seen before with pediatric brain tumor models, PDOX tumors of adult GBMs also displayed an increased growth rate (decreased survival times) starting from passage II in > 60% of the models and become stabilized on or after passage III (e.g., K023GBM and K014GBM) (Fig. 1a) in addition to approximately 20% models exhibiting stable growth rate from passage I up to passage V (e.g., K012GBM and K035GBM), or slightly slowed growth /prolonged survival times (e.g., K038). These data showed the inter-tumoral heterogeneity of GBM.

### PDOX tumors replicated the histopathological features of the original patient tumors

The morphological characteristics of the transplanted tumors, as examined by H&E staining, were well maintained in the corresponding xenograft tumors both cellularly and structurally (Additional file 1: Fig. S1). Histological features of GBM, including marked hypocellularity, nuclear atypia, necrosis, vascular proliferation and active proliferation, were maintained in the matching PDOX tumors (Fig. 1b). Immunohistochemical staining on glial marker GFAP, cell proliferation (Ki67), intermediate neurofilament vimentin (VMT), tumor suppressor gene p53 and micro blood vessels with Von Willebrand factor (VWF), and tumor cell mitochondria (MT) revealed a broadly similar pattern of staining positivity between PDOX tumors and their originating patient tumors (Fig. 1b; Additional file 1: Fig. S1). The IHC also detected inter-tumoral differences among the models with IC-K023GBM exhibiting the highest (> 60%) cell proliferation (Ki-67), IC-K024GBM, IC-K033GBM and IC-K038GBM expressing strong GFAP (> 90% cells with +++). P53 positivity was detected in nearly all the cells in 6/14 models (IC-A46GBM, IC-K012GBM, IC-K023GBM, IC0K024GBM, IC-K045GBM, and IC-V0914AA) indicating gene mutation. Intense endothelial proliferation was confirmed by VWF staining in IC-K014GBM, IC-K023GBM, IC-K045GBM, IC-K060GBM, IC-A128GBM, and IC-V0914AA (Fig. 1b; Additional file 1: Fig. S1). Using antibodies against MT and VMT [18, 40], which were human specific, we confirmed the human origin of PDOX tumors (Fig. 1b).

### GBM PDOX models were highly invasive

Diffuse invasion is one of the hallmarks of GBM and the primary cause of treatment failure. Animal models faithfully replicating this critical feature of GBM are highly desired. Examination of paraffin sections of whole mouse brains frequently reveal diffuse invasion into the contralateral hemispheres through corpus callosum in nearly all the models (e.g., IC-K-023GBM, IC-K-35GBM, IC-K014GBM, formation of satellite tumors resulting from CSF dissemination and massive hydrocephalus (e.g., IC-K024GBM and IC-A46GBM; Fig. 1). Microscopically, migration of tumor cells into surrounding normal tissues as single cells, cluster of cells, or follow myelinated fiber tracks were frequently seen (Additional file 1: Figs. S1, S2). To positively identify human GBM cells in mouse brains, we performed IHC with human specific antibodies against mitochondria (MT) and neurofilament vimentin that have been serially tested and validated in our previous reports [18, 20, 24, 27-29, 41] and detected the invasive cells, particularly those single cells that migrated deep into the normal mouse brains (Fig. 1b; Additional file 1: Fig. S1) together with tumor core cells.

### PDOX models replicated key molecular features of the originating GBM tumors

**Fig. 2:**
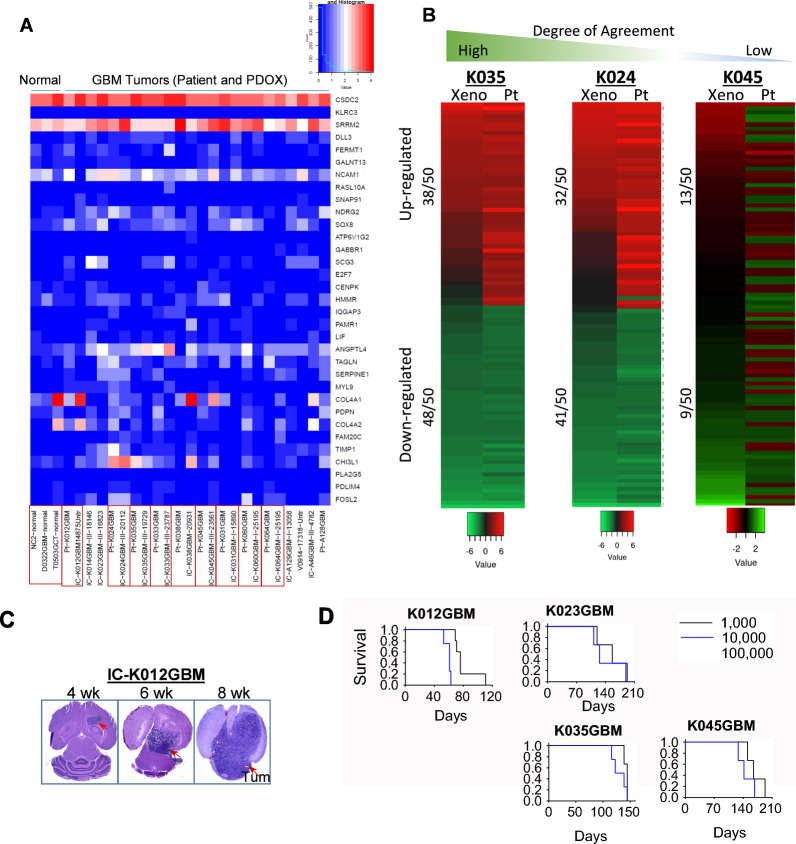
RNAseq analysis and in vivo tumor growth of PDOX models. **a **Hierarchical clustering of the top 33 differentially expressed genes in PDOX tumors along with their matching patient tumors. **b **Heatmap showing comparisons of differentially expressed genes between patient (***Pt***) and PDOX tumors (**Xeno**) in three models representing high levels of agreement in K035GBM, middle levels in K024GBM and low levels of K045GBM. **c **Time course in vivo tumor growth of ICK012GBM. **d **Log-ranks analysis showing the impact the implanted tumor cells (from 1,000 to 100,000) on the changes of animal survival times

Significant advances have been made in molecular subtyping of GBMs. As a model system, it is highly desired to represent a full spectrum of molecular subtypes. Using normal human cerebral tissue as reference, we performed RNAseq analysis of the PDOX tumors with their matching patient tumors (GEO access number: GSE207886) and identified a set of shared genes up- (CSDC2 and SRRM2) or down-regulated (e.g., DLL3, GALNT13, RASL10A and SOX8) in the tumors (Fig. 2a). To further determine if PDOX tumors replicated the patient tumor gene expression profiles, we excluded genes with zero counts in all samples (*n* = 10,051) and used the remaining 34,735 genes to determine the correlation coefficient in nine models in which their matching patient tumors were available. The Pearson Correlation ranged from 0.54 to 0.72. To determine if the differentially expressed genes of the patient tumors were maintained in the PDOX tumors, we selected the top 50 up- and down-regulated genes from each of the nine patient tumors and compared with their matching PDOX tumors. Preservation of the upregulated genes ranged from 26 (in IC-K012GBM) to 76% (in IC-K035GBM) (48.9% ± 19.6%); and of the down regulated genes from 18 (in IC-K045GBM) to 96% (in IC-K035GBM) (66% ± 45.1%; Fig. 2b; Table 2). These data indicated the need of target validation in future biological and preclinical drug testing in the selected animal models.

**Table 2:** Molecular sub-classification of patient and/or PDOX models using two different classification system

Sample ID	500 genes	840 genes	Correlation coefficient	Human cell %
*Matched pairs*				
Pt-K012GBM	Proneural/neural	Proneural/neural	0.56	100
IC-K012GBM-III	Proneural/neural	Proneural/neural		56
Pt-K031GBM	Proneural/neural	Proneural/neural	0.66	99.8
IC-K031GBM-I	Proneural/neural	Proneural/neural		92.8
Pt-K024GBM	Classical	Mesenchymal	0.67	100
IC-K024GBM-III	Classical	Mesenchymal		64
Pt-K060GBM	Classical	Mesenchymal	0.64	
IC-K060GBM-I	Classical	Proneural/neural		31.6
Pt-K064GBM	Classical	Mesenchymal	0.63	100
IC-K064GBM-I	Classical	Proneural/neural		n/d
*Not matched pairs*				
Pt-K033GBM	Classical	Mesenchymal	0.64	100
IC-K033GBM-III	Mesenchymal	Classical		51.9
Pt-K035GBM	Classical	Mesenchymal	0.72	100
IC-K035GBM-III	Mesenchymal	Classical		66
Pt-K038GBM	Proneural/neural	Proneural/neural	0.54	100
IC-K038GBM-III	Mesenchymal	Classical		48.1
Pt-K045GBM	Proneural/neural	Proneural/neural	0.54	100
IC-K045GBM-III	Classical	Mesenchymal		82
*PDOX model only*				
IC-A129GBM-I	Classical	Proneural/neural		n/d
IC-A46GBM-III	Proneural/neural	Proneural/neural		50
IC-K014GBM-III	Mesenchymal	Proneural/neural		40
IC-K023GBM-III	Proneural/neural	Proneural/neural		50
IC-V0914-III	Proneural/neural	Proneural/neural		93.7

To rule out the possibility of sample mis-match and cross contamination, we also performed DNA finger print analysis using 24 markers as we did previously [32]. All the xenograft models matched with their patient tumors (Additional file 1: Table S1). Since our models were diffusely invasive and the DNAs were extracted from bulk xenograft tumors, we reasoned that mouse cell DNAs were present in the xenograft samples. Using qPCR, we detected substantial amount of mouse DNA, ranging from 18 to 60% in every PDOX tumors (Table 2). The mouse cell contamination, even after filtration with universal mouse DNA sequences, may have contributed to the genetic “noise” and the suboptimal correlation.

### Three major subtypes of GBMs were represented in our panel of PDOX models

Many advances have also been made in molecular sub-classfications of GBM [30, 31, 42-44]. A total of 24 samples were available for RNA-seq: 9 PDOX models paired with original patient tumors (n = 18) and 6 additional patient GBM tumors or PDOX models (n = 6). We first classified the molecular subtypes of these 24 tumors using Verhaak’s original 840 gene set [30] and identified 3 PDOX models matched the original patient tumor subtype (Additional file 1: Fig. S3). We then used our recently published 500 gene set, which was a TCGA-derived gene-classifier that can be applied across different gene profiling platforms and population groups [31], to find 5 PDOX models matched the original molecular subtype of the patient tumor (Table 2; Additional file 1: Fig. S4). The differences of molecular subtyping of the remaining 4 pairs may have been caused by the mouse cell contamination, ranging from 7 to 68% (Table 2). Of the matched pairs (patient tumor and PDOX tumor), we have 2 proneural and 3 mesenchymal by the 500 gene classifier [31], and 2 proneural and 1 classical model by the 840 gene classification [30]. In total, of matched or unmatched specimens comprising of patient tumor/PDOX tumor, we have 6 proneural/neural, 1 classical and 6 mesenchymal PDOX models by 500 gene classifier (Table 2; Additional file 1: Fig. S4), and 8 proneural/neural, 5 classical and zero mesenchymal models (Additional file 1: Fig. S3**)** by Verhaak’s classifier. This panel of PDOX models should serve as a resourceful platform for GBM studies.

### Animal survival times were inversely correlated with the number of the implanted tumor cells

Having established the tumor formation with 1 × 10^5^ cells/mouse, we attempted to follow tumor growth in vivo by serial sections on whole mouse brains every 2–3 weeks post tumor implantation (Fig. 2c). We next examined if reducing cell numbers will maintain 100% tumorigenicity and if animal survival times will be altered and at what levels by engrafting 1 × 10^5^, 1 × 10^4^ and 1 × 10^3^ cells/mouse (n = 5) at passage III in 5 GBM models (Fig. 2d). In all 5 models, tumor formation was confirmed in all the mice, including those implanted with 1,000 cells. Analysis of animal survival times revealed a reverse correlation with the implanted tumor cell numbers (*P* < 0.05). The differences of survival times between 1 × 10^5^ and 1 × 10^4^ tumor cell groups were more pronounced than the differences between 1 × 10^4^ and 1 × 10^3^ tumor cells groups, despite the 10- fold reduction of the implanted tumor cells. When reducing the implanted cells form 1 × 10^5^ to 1 × 10^3^ (100-fold reduction), we only observed limited extension (less than 1 fold) of animal survival times. These results demonstrated the maintenance of tumorigenicity even with 1,000 tumor cells. It also revealed an important impact of the implanted cell numbers on the prolongation of animal survival time, which can potentially serve as a “standard curve” of tumor growth to help evaluate therapeutic efficacy of new therapies.

### SVV-001 infects and kills primary cultured GBM PDOX tumor cells

**Fig. 3:**
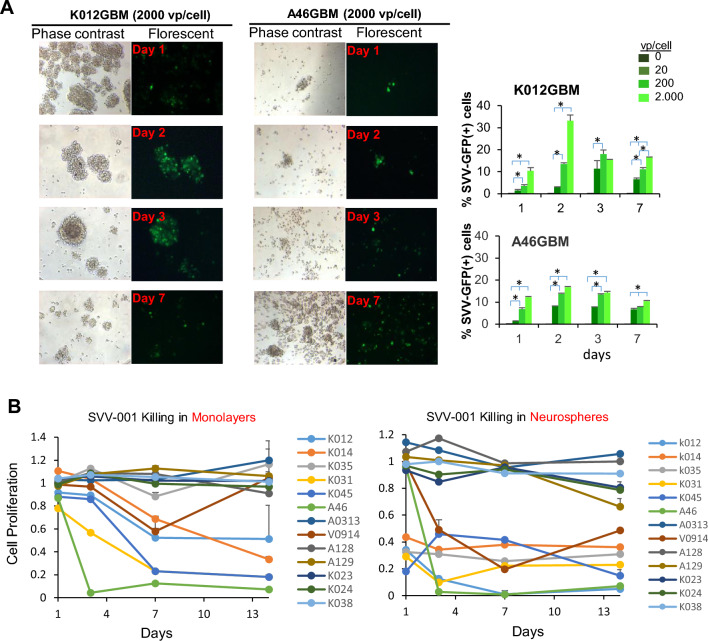
Infection and killing of GBM cells by SVV-001 in vitro. **a **Infection of neurosphere cultures derived from two GBM PDOX models. Tumor cells incubated with SVV-GFP from 20 to 2,000 viral particles/cells for 1 to 7 days were subjected to image detection of GFP+ cells (*left panel*) followed by quantitative analysis with FCM (*right panel*). *P<0.05 between the treated groups. **b** Time-course analysis of cell proliferation by SVV-001 (0.5 vp/cell) for 14 days. Cell viabilities in the treated cells were normalized to the untreated cells

Traditional FBS-based media and serum-free media supplemented with growth factors are shown to favor the growth of putative cancer stem cells (CSCs). Our previous study also showed that simultaneous suppression of cell proliferation of both monolayer and neurosphere cells in vitro can lead to significant extension of animal survival times in vivo [25]. The 3D structure of neurospheres may make them less vulnerable than monolayer culture, we therefore selected 2 neurosphere lines derived from 2 models (IC-K012GBM and IC-A46GB) to evaluate the infectibility of SVV-001 in vitro using SVV-GFP, a genetically modified SVV-001 with the same tropism but reduced infection rate (i.e., need > 100 fold viruses to achieve same infection rate as the parent virus) [10, 34, 35]. The advantage of SVV-GFP is that it enables easy visualization and quantitation of tumor infection. To compensate for the reduced infection rate, the ratios of viral particles/cell were increased. As shown in Fig. 3a, we detected positive infection that was time- and dose-dependent. SVV-GFP doses at 20 viral particle/cell was able to infect tumor cells as early as 24 h (Fig. 3a). To determine the cell killing activities, we treated primary monolayer and neurosphere cultures of GBM derived from 13 PDOX models of which their primary xenograft cells proliferate both as monolayer and neurospheres for at least 14 days with the parent SVV-001 in triplicates, ranging from 0.5 to 25 viral particles (VP)/cell, for 14 days. A reduction in cell viability (> 50%) was detected in 5 (38.5%) primary cultured xenograft tumor cells (derived from 4/9 proneural, 0/3 classic and 1/2 mesenchymal models) (Fig. 2b) and 7 (53.8%) neurosphere cultures derived from 5/9 proneural, 1/3 of classic and 1/2 mesenchymal models. These data showed broad anti-tumor activities of SVV-001 against different molecular types of GBMs.

### GBM cells expressing putative cancer stem markers are permissive to SVV-001 infection

**Fig. 4:**
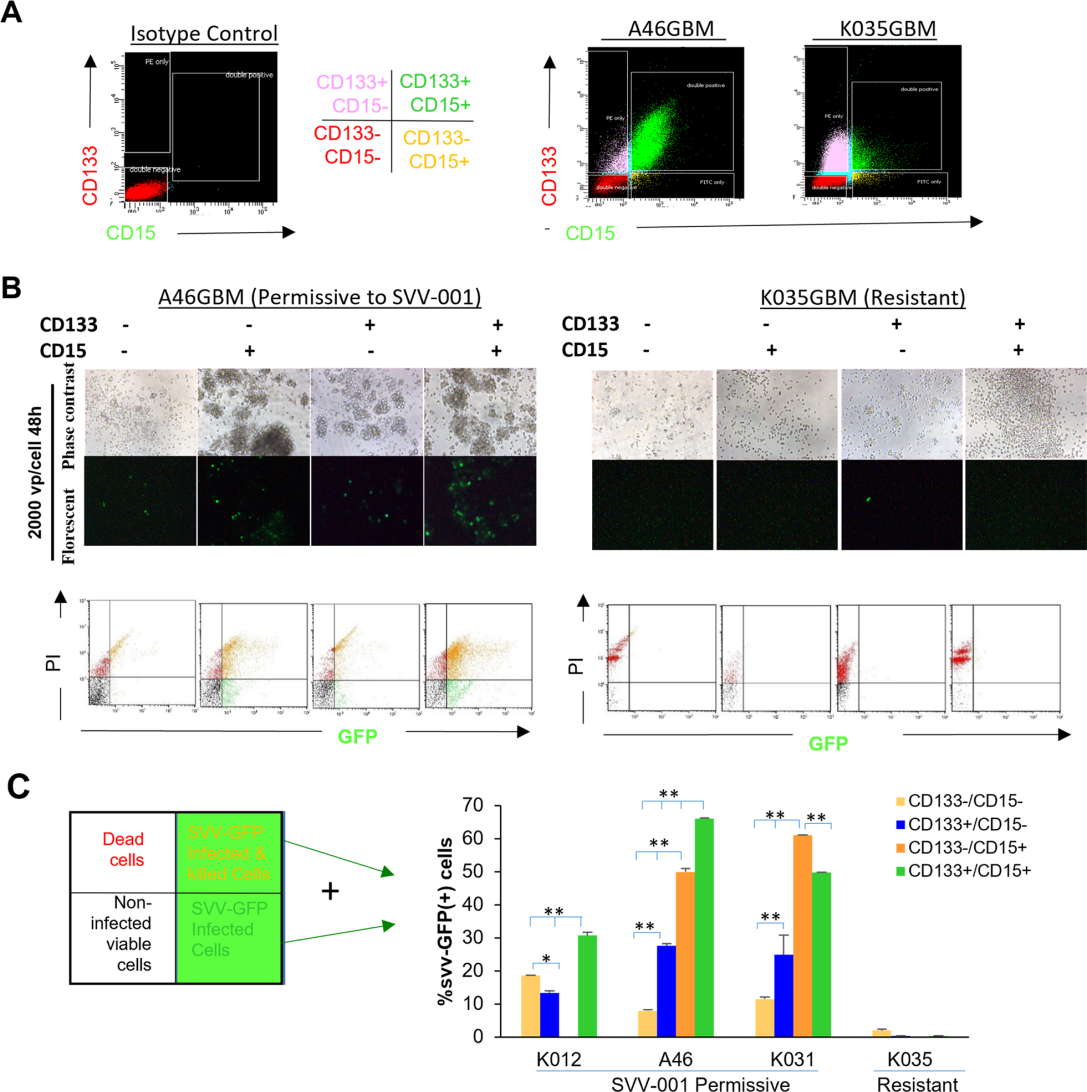
Infection of putative cancer stem cells of GBM in vitro. **a **Florescent cell sorting profiles of GBM cells after CD133 and CD15 dual staining. **b** Florescent and phase contrast images of SVV-GFP infection of the purified CD133 and/or CD15 positive GBM cells in a permissive model (A46GB) as compared with a resistant model (K035GBM). **c** Quantitative analysis of SVV-GFP infectivity in FACS purified CD133+ and/or CD15+ cells from 3 permissive GBM models and 1 resistant model. *P<0.05; **P<0.01

Poor prognosis of GBM is linked to rapid proliferation and cell heterogeneity, including glioma stem cells. Given the critical roles of CSCs in therapy resistance and tumor recurrence [45, 46], it is important to determine whether SVV-001 can eliminate adult GBM stem cells. Despite controversies of GBM CSCs, CD133 and CD15 has been successfully used to characterize and isolate at least a subpopulation of CSCs [47-49]. We performed double staining of CD133 and CD15 to examine the susceptibility of mono- and dual-positive GBM cells toward SVV-001. We used SVV-GFP, the genetically modified SVV-001 that has a lower infectivity compared with natural SVV-001 [34, 35] to infect FACS-purified CD133^+^/CD15^+^, CD133^+^ CD15^−^, CD133^−^CD15^+^and CD133^−^/CD15^−^ cells derived from 4 PDOX models (3 permissive and 1 resistant to SVV-001) (Fig. 4). In the 3 permissive models treated with SVV-GFP for 48 h, effective infection was observed in the CD133^+^/CD15^+^ cells, resulting in 30.7% positivity in IC-K012GBM, 49.7% in the IC-K031GBM and up to 66% in the IC-A46GBM (*P* < 0.05; Fig. 4a, c); whereas in the resistant tumors (IC-K035GBM), less than 5% SVV-GFP positivity was observed (*P* < 0.05; Fig. 4a–c) regardless of the expression status of CSC markers. The levels of SVV-GFP infectivity in the CD133^−^/CD15^−^ tumor cells were less than those observed in the corresponding CD133^+^/CD15^+^ cells (*P* < 0.05), with high-level GFP positivity found in the permissive tumors and low positivity in the resistant models (Fig. 4c). These data showed that SVV-GFP was capable of infecting CD133 and/or CD15 positive cells in permissive GBM models.

#### Intravenously injected SVV-001 can pass through the BBB to infect PDOX glioma cells

**Fig. 5:**
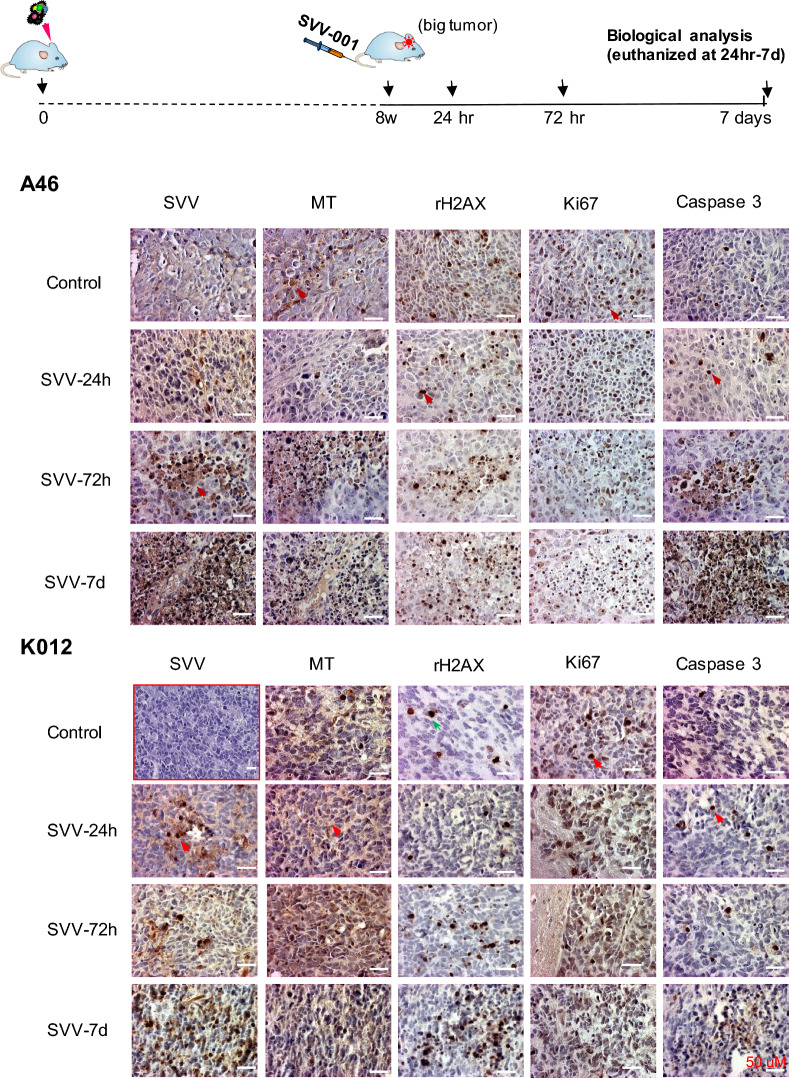
Time-course immunohistochemical analysis of PDOX tumors treated with SVV-001. The implanted GBM cells from two GBM models IC-A46GBM (***A46***) and IC-K012GBM (**K012**) were allowed to grow for 8 weeks to form xenograft tumors before receiving a single tail vein injection of SVV-001 (1 × 10^11^vp/kg) (***top panel***) and examined for *in vivo *SVV-001 (SVV) infection, DNA damage (rH2AX), cell proliferation (Ki67), apoptosis (Caspase 3) as well as human-mitochondria (MT) 24 hr—7 days post SVV-001 administration. An image of H&E staining of IC-K-012GBM (*highlighted with red boarder*) was included to show the histology of untreated tumor core. (Scale bar: 50 µM)

One of the advantages of SVV-001 relative to many other oncolytic viruses is that it can be administered through tail vein injection and pass through the blood brain barrier (BBB) while sparing normal mouse brain cells [11, 35, 50]. To examine its in vivo infection activities against GBM cells, IHC was performed on whole mouse brain paraffin sections to examine the time-course changes of SVV-001 infection at 24 h, 48 h, 72 h and 7 days post a single *i.v.* injection. A time-dependent increase of SVV-001 infection was detected. Isolated and small patches of SVV-001 positive tumor cells were detected as early as 24 h post SVV-001 injection in both GBM models (IC-A46GBM and IC-K012GBM). The infected cells increase over time, resulting in large areas of dead GBM cells stained positive with Caspase3 and significant loss of cell proliferation (Ki67) by day 7 (Fig. 5). DNA damage as detected by rH2AX was also seen and paralleled the expansion of the infected tumor cells (Fig. 5). SVV-001 is a replication competent oncolytic virus [7, 10, 35]. Intra-cellular replication, subsequent lysis of the target cells and the release of mature viruses may have contributed to the rapid and amplified infection of neighboring tumor cells characterized by cytoplasmatic inclusion, condensation and fragmentation of nucleus (Additional file 1: Fig. S5A) [35]. In addition to tumor core cells, invasive GBM cells, including single invasive cells, were also infected (Additional file 1: Fig. S5B). Similar to our previous observations in pediatric GBM medulloblastomas PDOX mouse models [11, 35, 50], SVV-001 did not infect adjacent normal mouse brain cells, confirming the safety profile of SVV-001.

### SVV-001 treatment with and without radiation significantly prolongs animal survival times

**Fig. 6:**
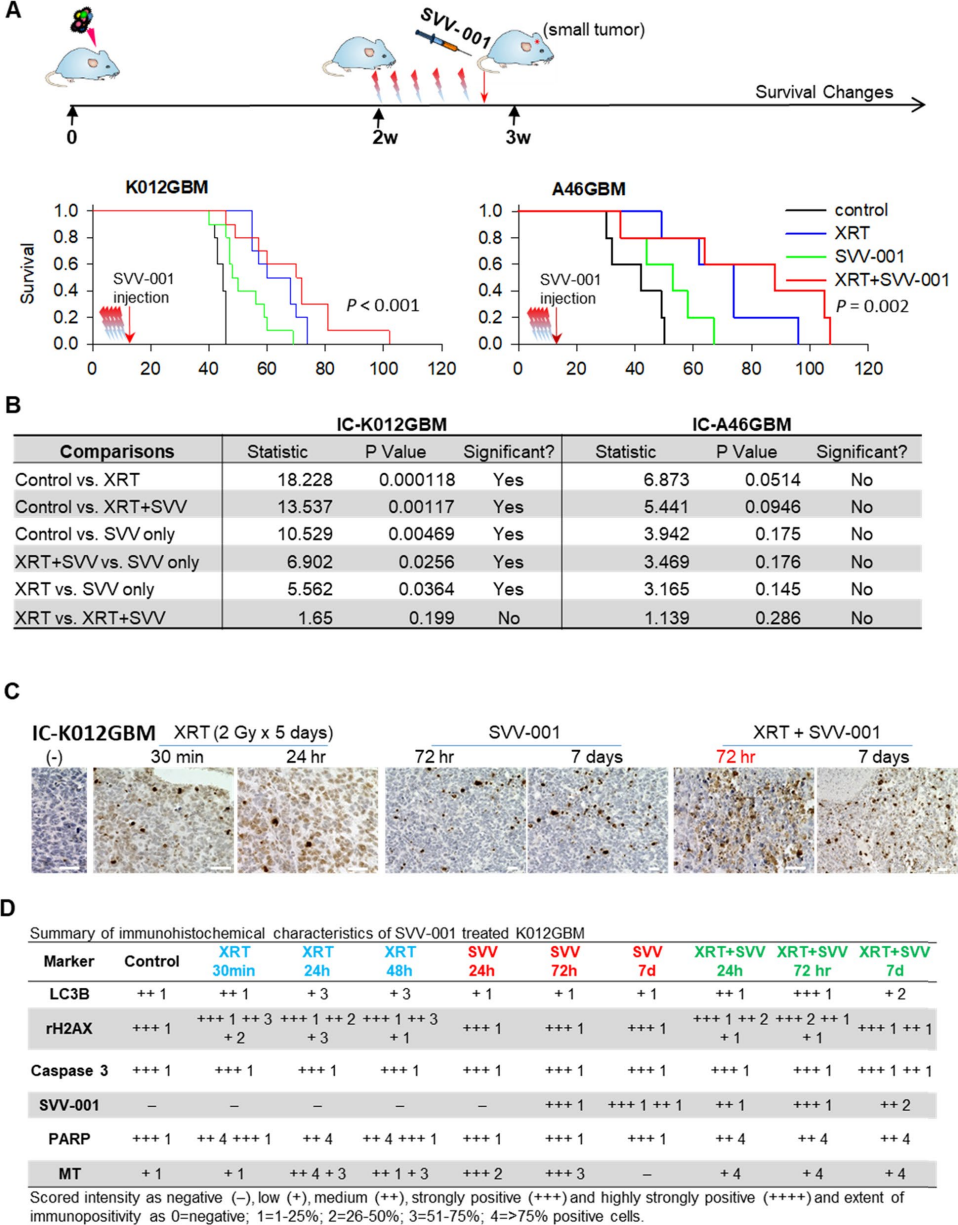
In vivo therapeutic efficacy of SVV-001 acting alone and in combination with fractionated radiation. **a** Treatment scheme and log-rank analysis of animal survival times. Radiation was administered at 2 Gy/day × 5 days after which SVV-001 (1 × 10^11^vp/cell) was administered via tail vein injection. Changes of animal survival times were compared via log-ranks analysis. Median survival times in IC-K012GBM were 46 days in control group, 50 days in SVV-001 only group, 59 days in XRT only, and 69 days in the combination; whereas in IC-A46GBM, they were 42 days in control group, 53 days in SVV-001 only, 74 days in XRT only, and 88 days in the combination. **b** Summary of pairwise comparison of the treated groups using Holm–Sidak method. *P* < 0.05 is significant. **c, d** Images and summary of time-course IHC analysis of DNA damage (rH2AX) in PDOX tumors of IC-K012GBM following XRT and/or SVV-001 treatment. (Scale bar: 50 µM)

Single agent in cancer treatment often exhibits limited efficacy. To fully evaluate the therapeutic efficacy of SVV-001, we treated two permissive GBM models with SVV-001 acting alone and in combination with fractionated radiations, a key component of standard therapies. Forty mice were orthotopically implanted with IC-K012GBM and IC-A46GBM and divided into four groups (n = 10 per group): control, SVV-001 only, XRT only, and XRT + SVV-001. Two weeks post-tumor cell implantation (when solid tumor ~ 1–2 mm in diameter were formed), radiation was administered locally at 2 Gy/day for 5 days, after which SVV-001 (1 × 10^11^ vp/kg) was administered with single tail vein injection (Fig. 6a). Changes of animal survival times were performed through log-rank analysis followed by post hoc pairwise multiple comparison procedures using Holm–Sidak method (Fig. 6b). Compared with the untreated control, SVV-001 prolonged the median animal survival times from 45 to 50 days (22.2% increase) (*P* = 0.0026) in IC-K012GBM, and from 42 to 53 days (26.2%) (*P* = 0.002) in IC-A46GBM. Mice treated with XRT in IC-K012GBM also exhibited significant extension of survival times better than the SVV-001 alone (*P* < 0.01). Combining SVV-001 and XRT further extended the survival time in > 50% of the mice in IC-K012GBM model beyond SVV-001 acting alone (*P* = 0.0256), although the overall extension was not statistically significant when compared with XRT only group (*P* > 0.05) (Fig. 6b). Pairwise comparison in IC-A46GBM did not reveal significant differences (Fig. 6b). These data demonstrated the therapeutic efficacy of SVV-001 and suggested that optimization of timing, scheduling and frequency of the combination with XRT is needed to potentially further improve survival times.

To understand the mechanisms of action of the combined therapy, we examined the time course changes of DNA damage in mice treated with SVV-001, XRT and the combined SVV-001 and XRT via IHC staining of rH2AX, a marker of DNA breaks. As anticipated, XRT at 2 Gy/day x 5 days resulted in rH2Ax positivity starting from 30 min post the last treatment and increased till 24 and 48 h (Fig. 6c, d). SVV-001 also induced DNA damage (strong +++ of rH2AX) in a small fraction (< 25%) of tumor cells, starting from 72 h and lasted to 7 days post virus injection (Figs. 5, 6b). Combining XRT and SVV-001 increased the cells with strong (+++) positivity from < 25% in XRT and SVV-001 groups to ~ 50% at 72 h with increased cell death by day 7. These data revealed a novel activity of SVV-001 in causing DNA breaks, which in turn may have additively enhanced the radiation induced DNA damages in a subset of tumor cells, leading to increased tumor cell killing.

### Expression of ANTRX1 in predicating GBM permissiveness toward SVV-001

**Fig. 7:**
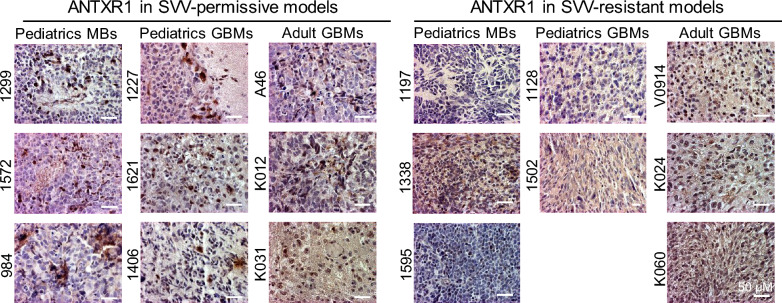
Examination of ANTRX1 protein expression in a panel of brain tumors permissive to (n = 9, *left panel*) or resistant (n = 8, *right panel*) to SVV-01 to correlate the expression of ANTRX1 with SVV-001 responsiveness

Given the differential responses toward SVV-001 in adult GBM tumors, it is highly desired to develop diagnostic markers for patient selection. We and others have made significant efforts to identify the molecules that mediate the selective infection, i.e., tropism, of SVV-001 to a subset of human cancers while sparing nearly all normal cells of human being [7, 10, 33, 35]. In addition to putative neuro-endocrine markers [50], ratio of NEUROD1 to ASCL1 [33], and sialic acids (in pediatric GBMs as we analyzed) [10], Anthrax Toxin Receptor 1 (ANTRX1) was also found to be a candidate molecular receptor of SVV-001 [51, 52]. To determine the role of ANTRX1 in mediating SVV-001 infection in GBM cells, we analyzed the expression of ANTRX1 in a panel of 17 PDOX models to correlate it with their SVV-001 responsiveness. In the 9 PDOX models (3 medulloblastomas [35], 3 pediatric GBMs [10] and 3 adult GBMs) that were previously [10, 11] and currently shown to be responsive to SVV-001, positive staining of ANTRX1 was detected but in a small fraction of tumor cells (< 25%); whereas in the 8 PDOX models resistant to SVV-001, only 4 models (2/3 medulloblastomas [35], 1/2 pediatric GBMs [10] and 0/3 adult GBMs) appeared to be ANTRX1 negative (Fig. 7). Since tumor cells from ICb-1299 MB and ICb-1572 MB could be infected > 95% in our previous studies [35], the existence of large numbers of ANTRX1 negative tumor cells seemed to suggest the existence of additional molecule(s) that determines tumor susceptibility to SVV-001 infection [10].

## Discussion

Clinically relevant and molecularly accurate animal models that represent a broad spectrum of GBM subtypes can facilitate biological studies and preclinical drug testing. In the current study, we have successful developed 17 PDOX models of adult GBM by direct implantation of surgical samples into the matching locations in mouse brains. Detailed pathological and molecular characterization demonstrated the replication of the biology of the original patient tumors and represented the 3 molecular subtypes of GBM. Using this set of transplantable and highly invasive GBM animal models, we tested the therapeutic efficacy of an oncolytic virus (through tail vein injection) in combination with fractionated radiation therapy.

Over the past years, PDOX models (or orthotopic PDX) has gained significant recognition as one of the preferred model systems for understanding tumor biology and for developing therapeutic interventions [16, 18-22, 25, 27, 29, 40, 53, 54]. Orthotopic implantation into the mouse brain is thought to provide the appropriate microenvironment for brain tumors [53]. With an optimized logistics, standardized protocols, and expertise from different fields (surgery, molecular biology and animal care), we established a protocol for orthotopic implantation, which resulted in tumor take rate of 73.9%. Histopathological examination revealed faithful replication of the pathological features of the patient tumors, and the maintenance of the diffuse invasion that is characteristic of GBM. Direct comparison of gene expression profiles with RNAseq confirmed preservation of key differentially expressed genes and revealed some discrepancies between PDOX tumors and matching patient tumors, which is significantly different from our previous findings in pediatric ependymoma and medulloblastoma models [19, 55]. Contamination of mouse cells resulted from the significantly elevated (i.e., compared with ependymoma and medulloblastoma) and diffuse invasion of GBM PDOX tumors may be the primary cause. Compared with array-gene expression profiling that we previously used in pediatric brain tumors, the increased sensitivity of RNAseq to mouse cell contamination may have also contributed to the decreased correlation between patient and PDOX tumors, despite our efforts of digital filtering of mouse sequences. Our data of DNA fingerprint confirmed the identity match between the patient tumors and their matching PDOX model and provided important information for model tracking and validation in the future.

One of the advances in GBM diagnosis is the development of molecular subclassification system. Using an established 840 gene system and a 500 gene sub-classifier strategies, we discovered molecular-match in 5/8 models that had originating patient tumors. The difficulties of completely filtering out mouse RNA sequences combined with the evolution of PDOX tumors during serial in vivo subtransplanations (to passage III) may have played a role in the models that were subclassified differently from their originating patient tumors.

The PDOX models developed in this study provided an opportunity to test new therapies for GBM. As an oncolytic virus, SVV-001 has displayed safety profile in Phase I clinical trials [56], can be systematically administered through *i.v.* injection and can pass through the BBB [10, 35], making it an attractive therapy for GBM. In the current study, SVV-001 exhibited potent, rapid, and selective killing of GBM tumor cells, including CSCs, in vitro in a subset of the 13 PDOX-derived GBM cells. A single tail vein injection of SVV-001 also led to effective infection and killing of intra-cerebral GBM xenograft cells while sparing the normal mouse brain cells. Successful infection of the invasive tumor cells, as we detected in medulloblastoma and pediatric glioma models [10, 35], provided an added advantage of SVV-001 for GBMs.

Although SVV-001 as single agent with single tail vein injection led to significant extension of animal survival times in both PDOX models tested, intra-tumoral heterogeneity was noted as GBM cells resistant to SVV-001 was still present. Combining SVV-001 with clinically relevant fractionated radiation showed an exciting trend of further extension of animal survival times beyond radiation alone, indicating the potential of additive effects. Our novel finding of SVV-001’s capacity in causing DNA damage in very exciting. It may have contributed to radiation induced DNA breaks and promoted tumor cell killing. Successful fine-tuning of the dose, frequency, schedule and relative timing of SVV-010 and radiation therapy in the future may further improve the efficacy. Since SVV-001 is a replication competent virus [7, 9, 10, 35, 50], ionizing radiation can cause mutations of the virus potentially resulting in dramatic changes of virus properties (changes of host cells, replication rate and cell killing efficiency, ect). It is therefore important to avoid radiating any animals carrying live SVV-001 virus. Administering SVV-001 after the completion of fractionated radiation therapy can safely prevent such accidents. As for the mechanisms of combined therapy, previous studies suggested the inhibition of DNA repair pathway by oncolytic virus mediated the cell killing for the combination with radiation therapy [57]. Identifying the key enzymes and pathways affected by SVV-001, particularly following an optimized and effective combination with radiation, should help a rationally designed combination. Recent advances of oncolytic virus immunotherapy [58, 59] also suggested an opportunity for SVV-001 in GBMs, although the host animals need to be immunocompetent for preclinical testing.

The ability of a given virus to productively infect a particular cell (i.e., cellular tropism) is frequently determined by receptor(s) [60, 61]. Many efforts have been invested to identify the receptor of SVV-001 [10, 50] and ATRX1 is one of the recently recognized molecules a candidate receptor for SVV-001 [51, 62]. By including a panel of 17 PDOX models of brain tumor that were shown to be responsive or resistant to SVV-001both in vitro and in vivo, we confirmed the positive staining of ATRX1 in the 9 responsive tumors. However, the relatively low positivity (< 25%) of ANTRX1 protein in the responsive tumors and the existence of positive cells in the resistant tumors suggested that additional studies are needed to validate the role of ANTRX1 as a receptor of SVV-001 because viral cellular tropism can be affected by various host innate immune anti-viral cytokines, including the interferons and tumor necrosis factor, as well [63]. Additionally, tumor endothelia marker 8 (TEM8) has also been revealed to mediate cellular entry of Seneca Valley Virus [64], providing a new candidate molecule to explore in the near future.

## Conclusion

We established a novel panel of 17 PDOX models of adult GBM and 1 anaplastic astrocytoma that replicated key pathological phenotypes and genetic abnormalities of the original patient tumors and represented three different molecular subtypes. These models should serve as a resourceful platform for biological and preclinical studies of GBM. Systematic treatment with an oncolytic virus, SVV-001, through tail vein injection significantly prolonged animal survival times and displayed a trend of further extension of survival times when combined with fractionated radiation therapy. Our findings support extended studies on SVV-001 in combination with standard therapy and/or immunotherapies for patients with GBM.

## Supplementary Information

**Additional File 1: Figures S1–S5.**

## Data Availability

Animal models will be distributed freely with fully executed material transfer agreement. RNAseq data have been deposited to public database GEO (access number: GSE207886).

## Abbreviations

GBM: Glioblastoma
PDOX: Patient derived orthotopic xenograft
SVV-001: Seneca Valley Virus
XRT: Radiation
ANTRX1: Anthrax Toxin Receptor 1
