# Supplementary material for: Targeting GBM with an Oncolytic Picornavirus SVV-001 alone and in combination with fractionated Radiation in a Novel Panel of Orthotopic PDX models
Source: J Transl Med. 2023 Jul 6;21:444. doi: 10.1186/s12967-023-04237-w (PMC10324131; doi:10.1186/s12967-023-04237-w)
Supplement: Supplementary file 1 — Additional File 1: Figures S1–S5. [file 12967_2023_4237_MOESM1_ESM.docx]

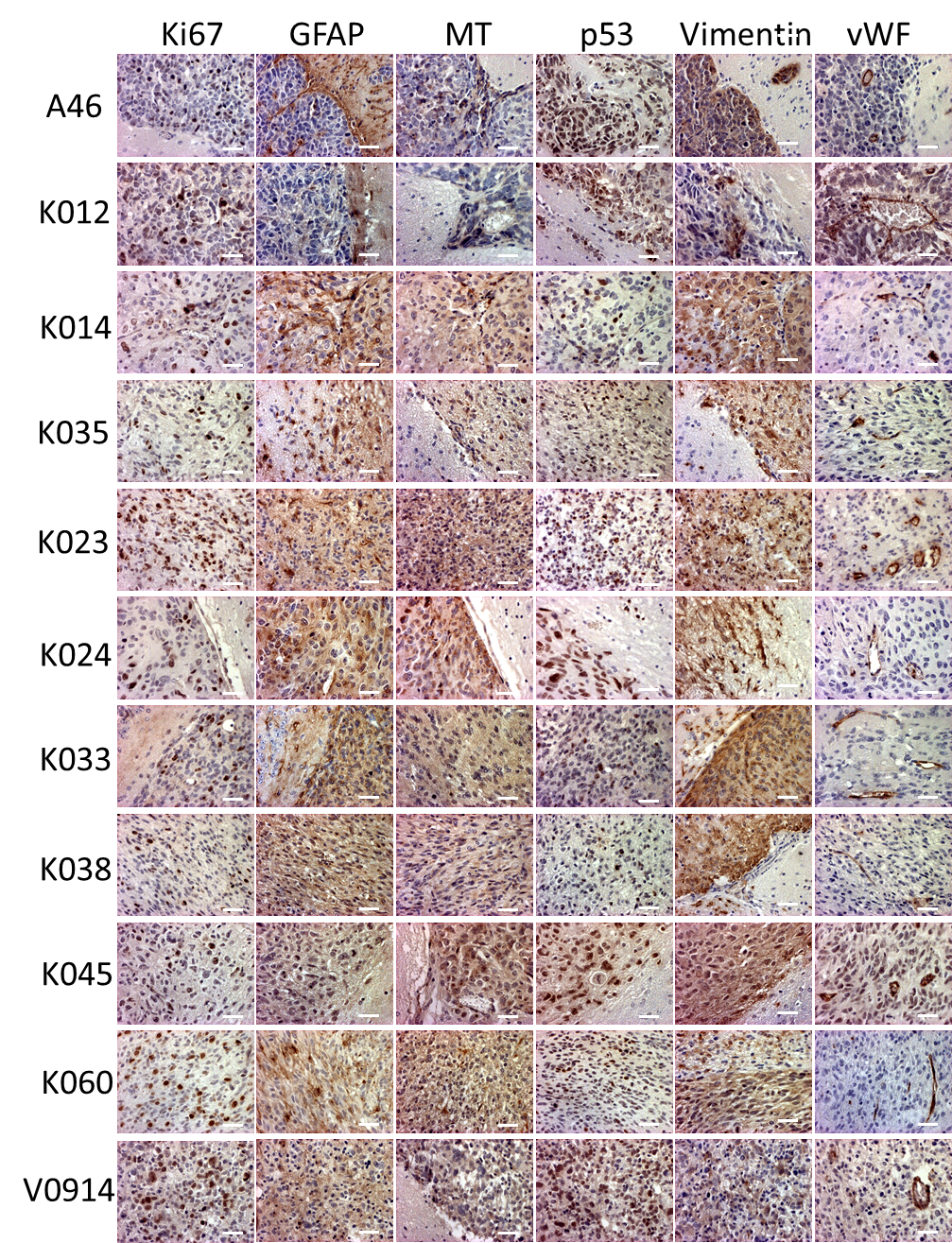


**Figure S1.** Representative images of IHC staining of markers of cell proliferation (Ki67), glial differentiation (GFAP), human-specific mitochondria (MT), tumor suppressor gene P53, human specific intermediate neurofilament vimentin (VMT), and mouse-specific blood endothelial marker von Willebrand Factor (vWF) in 11 PDOX models of GBM (from A46 to K060) and an anaplastic astrocytoma model (V0914).


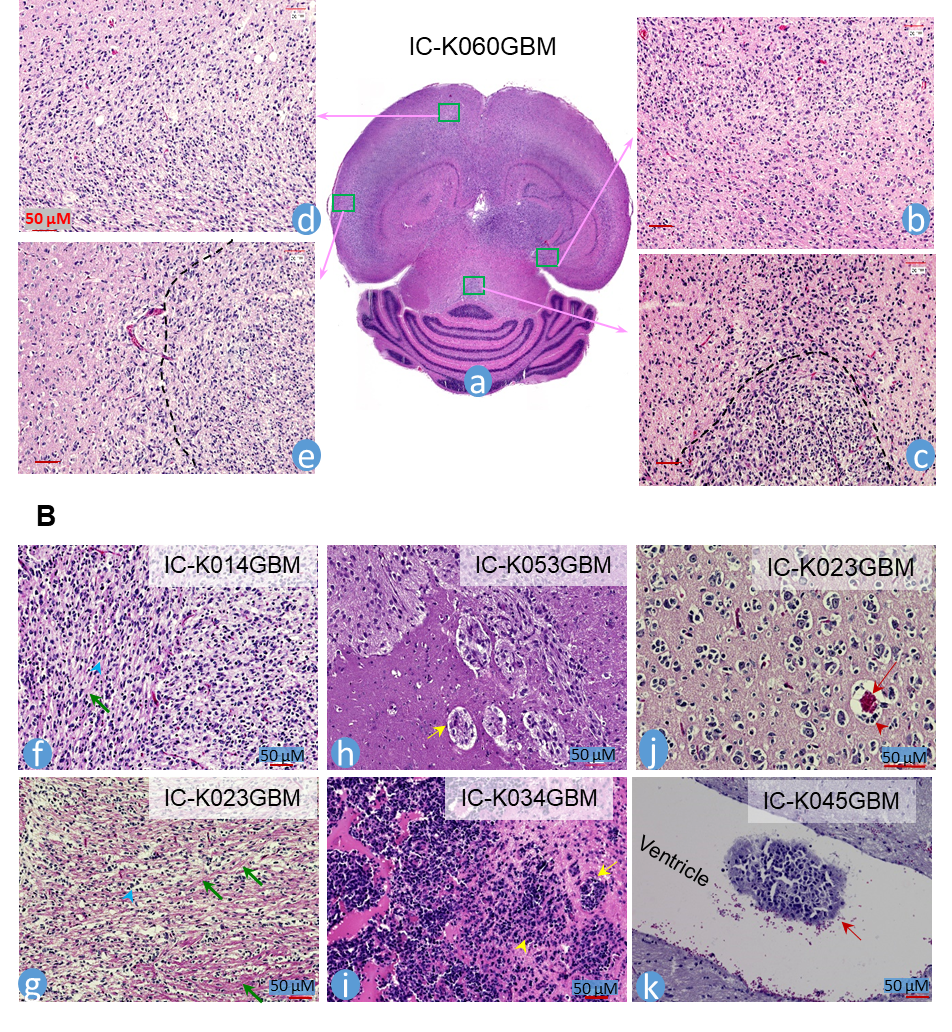


**Figure S2.** Representative images showing the diffuse invasion of GBM tumor cells. **A.** Sections of H&E staining in IC-K060GBM displaying GBM cell invading (*a*) into the neighboring tissue (*b*), to the 4th ventricle via CSF (*c*), and to the opposite side of the brain (*d, e*). **B.** Modes of GBM invasion in mouse brains, including migration along neural fibers (*f,g*), as clusters of cells (*h*), as group tumor cells (*arrowhead*) following blood vessels (*arrow*) (*j*), along collosum, as waves of tumor cells (*i*) and spread through CSF (*k*). Magnification: 40 x (*j*) and 20x (*b* to *i*, and *k*). (Scale bar: 50 µµM).


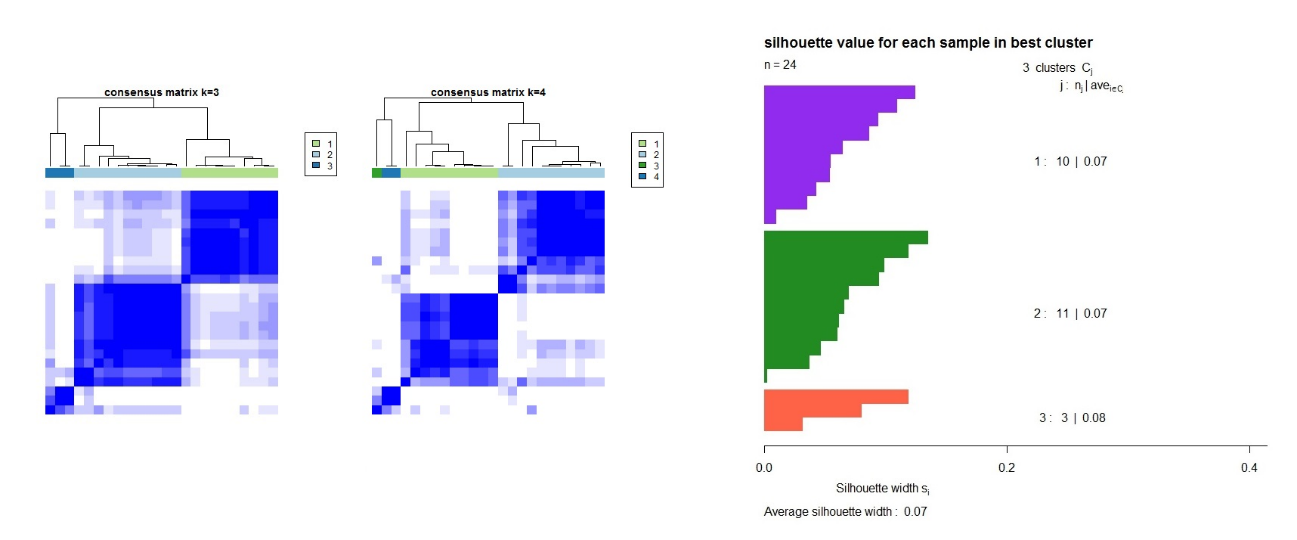

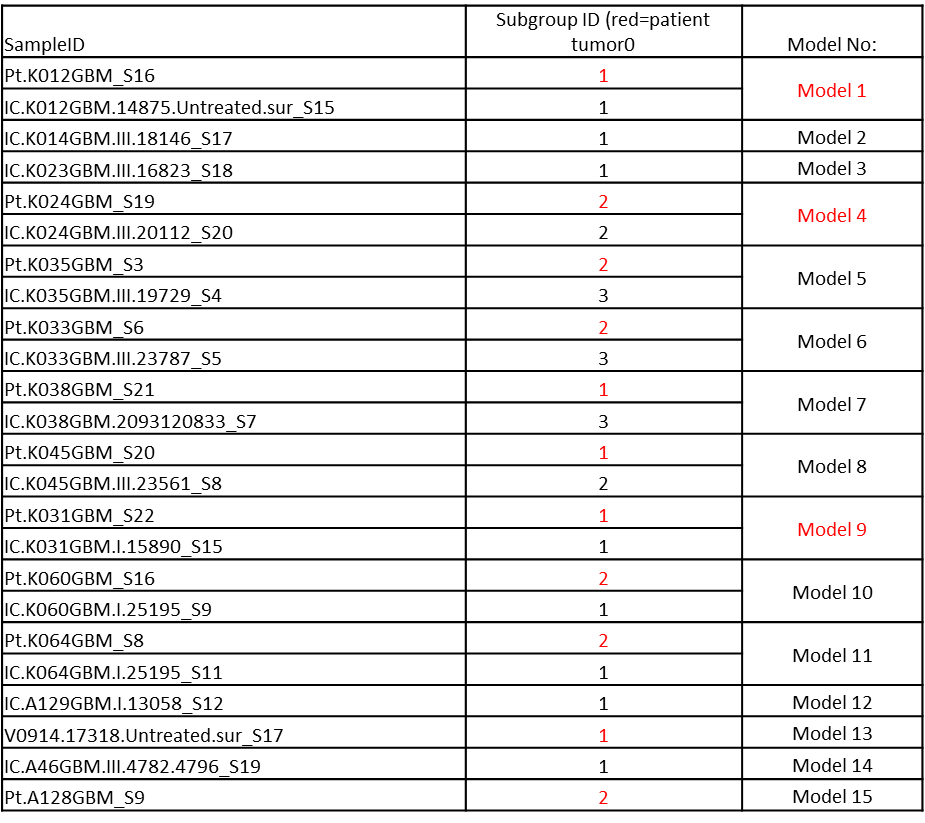


**A**

**B**

**C**

**D**

**Figure S3.** Application of Verhaak’s 840 gene-set (Verhaak, et al) to determine GBM subtype. Using RNA-seq data (non-normalized) of 24 tumor samples in our study cohort, (a) consensus hierarchical clustering plots and (b) silhouette plot, using Verhaak’s 840 gene-set (k=3), (c) (k=4), revealed 3 distinct clusters. (d) A total of 13 PDOX tumors are classified into Proneural/Neural (Subtype 1), Classical (Subtype 2) and Mesenchymal (Subtype 3). Model 15 are patient tumor. Subtype identity of 3 pairs of PDOX and original patient tumor pairs fully matched in Models 1, 4 and 9 ( highlighted in *red*).


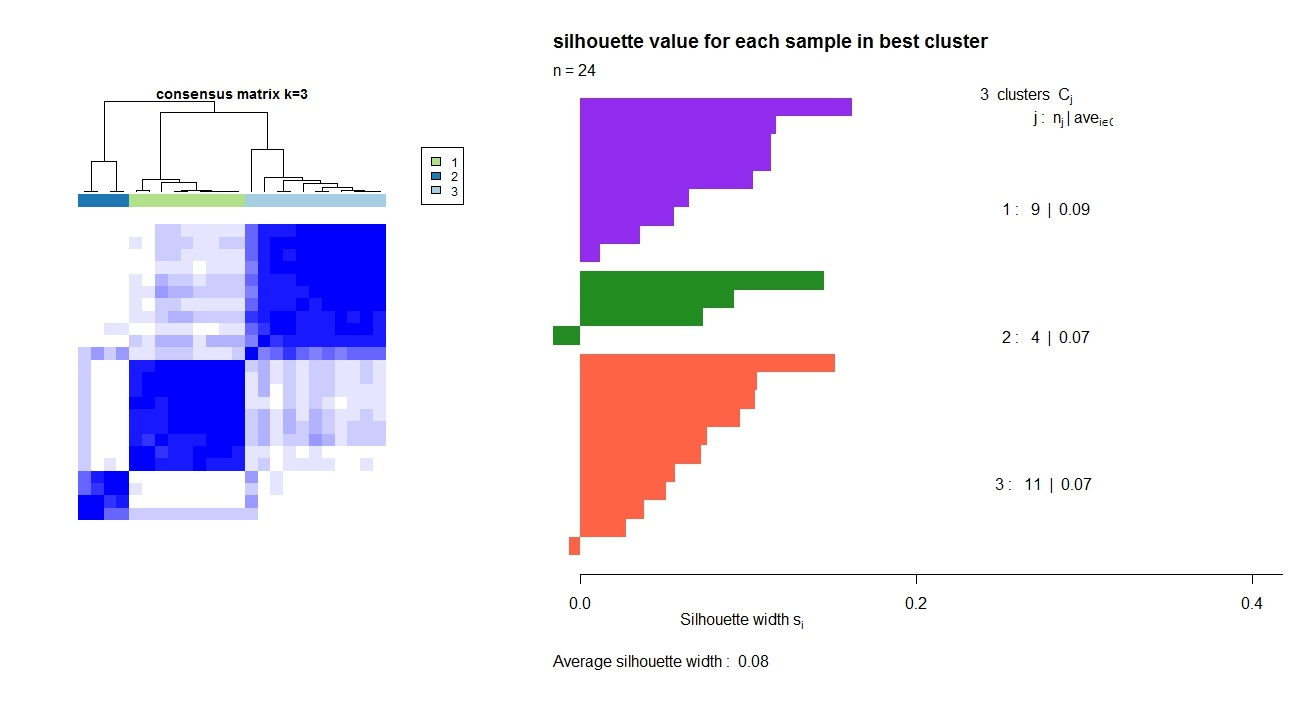

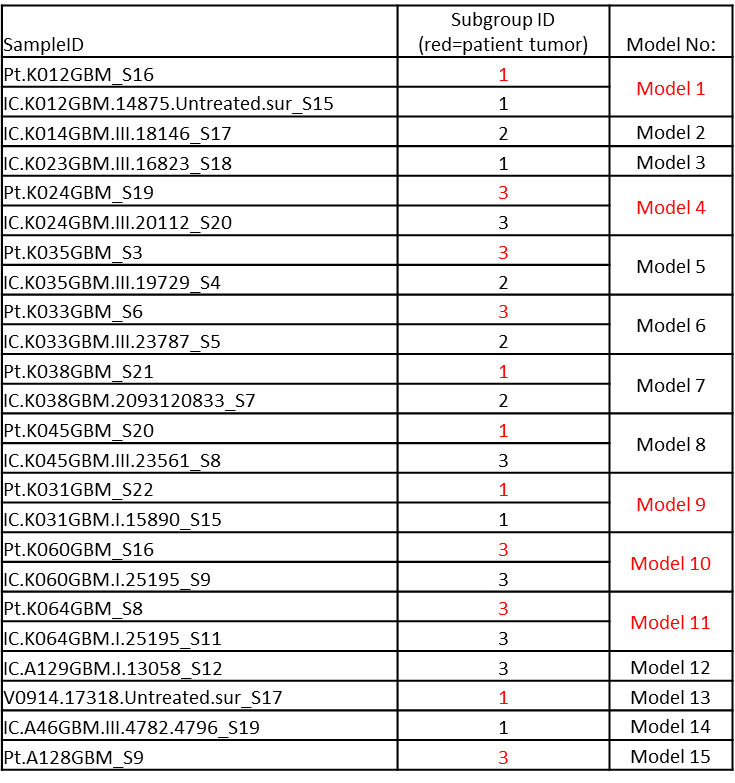


**A B**

**C**

**Figure S4.** Application of our previously published TCGA-derived 500 gene-classifier (Teo, et al 2019) to determine GBM-subtypes. Using RNA-seq data (non-normalized) of 24 tumor samples in our study cohort, (A) consensus hierarchical clustering plots and (B) silhouette plot, using our 500-gene classifier (k=3), revealed 3 distinct clusters. (C) A total of 13 PDOX tumors are classified into Proneural/Neural (Subtype 1), Classical (Subtype 2) and Mesenchymal (Subtype 3). Model 15 is patient tumor. Subtype identity of 5 pairs of PDOX and original patient tumor pairs fully matched in Models 1, 4, 9, 10 and 11 (highlighted in *red*).


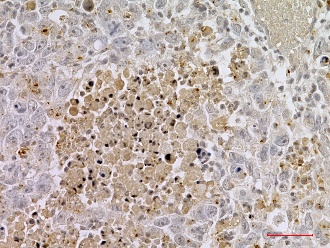

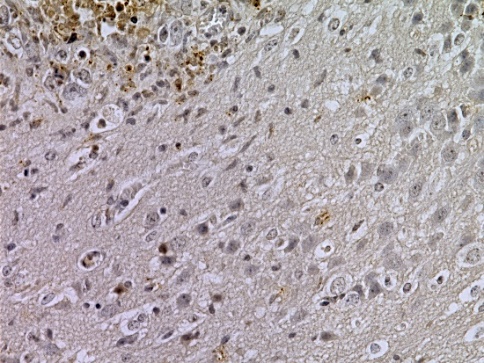

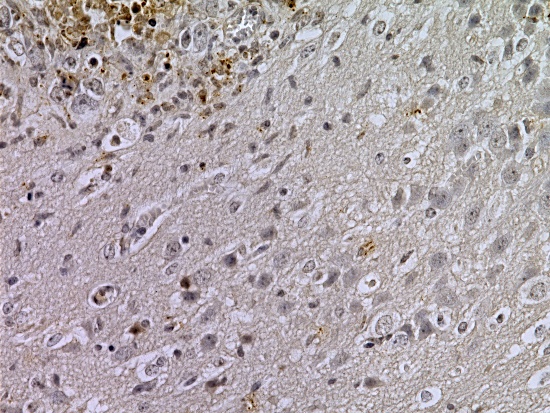

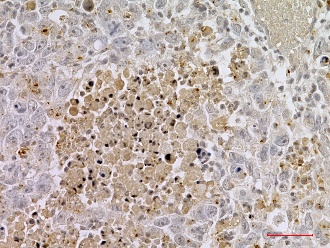


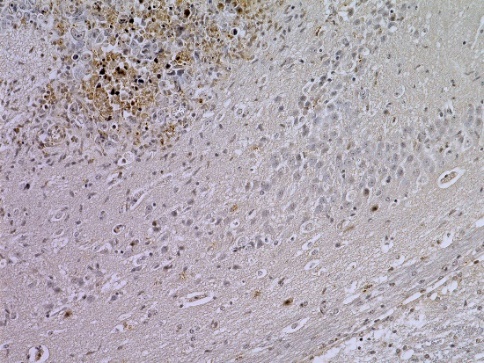


**A**

**B**

Tumor Core

Invasive Front

**Figure S5.** SVV-001 infection of GBM tumor cells in vivo in PDOX model IC-A46GBM 72 hr post i.v. injection. A. Representative image showing the cytoplastic inclusion and fragmentation of tumor cell nucleus (*arrowhead*) . B. Successful infection of GBM cells migrated into normal mouse brains (*arrow*).

50 nM
